# Myoelectric signal from below the level of spinal cord injury as a command source for an implanted upper extremity neuroprosthesis - a case report

**DOI:** 10.1186/s12984-019-0571-3

**Published:** 2019-08-02

**Authors:** Elizabeth Heald, Kevin Kilgore, Ronald Hart, Christa Moss, P. Hunter Peckham

**Affiliations:** 1 Dept. of Biomedical Engineering, Case Western Reserve University, 10900 Euclid Avenue, Wickenden Building, Cleveland, OH 44106 USA; 2 Louis Stokes Veterans Affairs Medical Center, Cleveland, OH USA; 3 MetroHealth Medical Center, Cleveland, OH USA

**Keywords:** Spinal cord injury, Neuroprosthesis, Myoelectric control, EMG, Functional restoration, Functional electrical stimulation (FES)

## Abstract

Implanted motor neuroprostheses offer significant restoration of function for individuals with spinal cord injury. Providing adequate user control for these devices is a challenge but is crucial for successful performance. Electromyographic (EMG) signals can serve as effective control sources, but the number of above-injury muscles suitable to provide EMG-based control signals is very limited. Previous work has shown the presence of below-injury volitional myoelectric signals even in subjects diagnosed with motor complete spinal cord injury. In this case report, we present a demonstration of a hand grasp neuroprosthesis being controlled by a user with a C6 level, motor complete injury through EMG signals from their toe flexor. These signals were successfully translated into a functional grasp output, which performed similarly to the participant’s usual shoulder position control in a grasp-release functional test. This proof-of-concept demonstrates the potential for below-injury myoelectric activity to serve as a novel form of neuroprosthesis control.

## Introduction

Neuroprostheses (NPs) are implantable devices that utilize small electrical currents to activate peripheral motor nerves, resulting in controlled contraction of paralyzed muscles. These devices may provide the most promising method for significant gain in hand and arm function for cervical level spinal cord injury (SCI) [1]. Currently, the preferred method to control implanted NPs is to use electromyographic (EMG) signals from muscles above the spinal cord lesion that remain under volitional control [2]. However, many SCI cases have a very limited number of above-injury muscles available for use as command signals, thus limiting the number, complexity, and efficiency of functions which can be effectively provided to the NP user. Identifying additional control sources would have a significant impact on the function that could be restored to these users.

In previous studies, EMG recordings from lower extremities of subjects with motor complete SCI indicated that the majority showed some degree of volitional activity in at least one muscle [3, 4]. If descending motor commands can be volitionally and reliably produced, the EMG generated may be used as a command signal, even if it is too small to produce discernable joint movement. Utilizing these signals as command inputs could provide innovative control options enabling improved performance in current NP systems. The purpose of this experiment was to demonstrate feasibility of using below-injury myoelectric activity as a command signal for an upper extremity neuroprosthesis.

## Methods

**Fig. 1:**
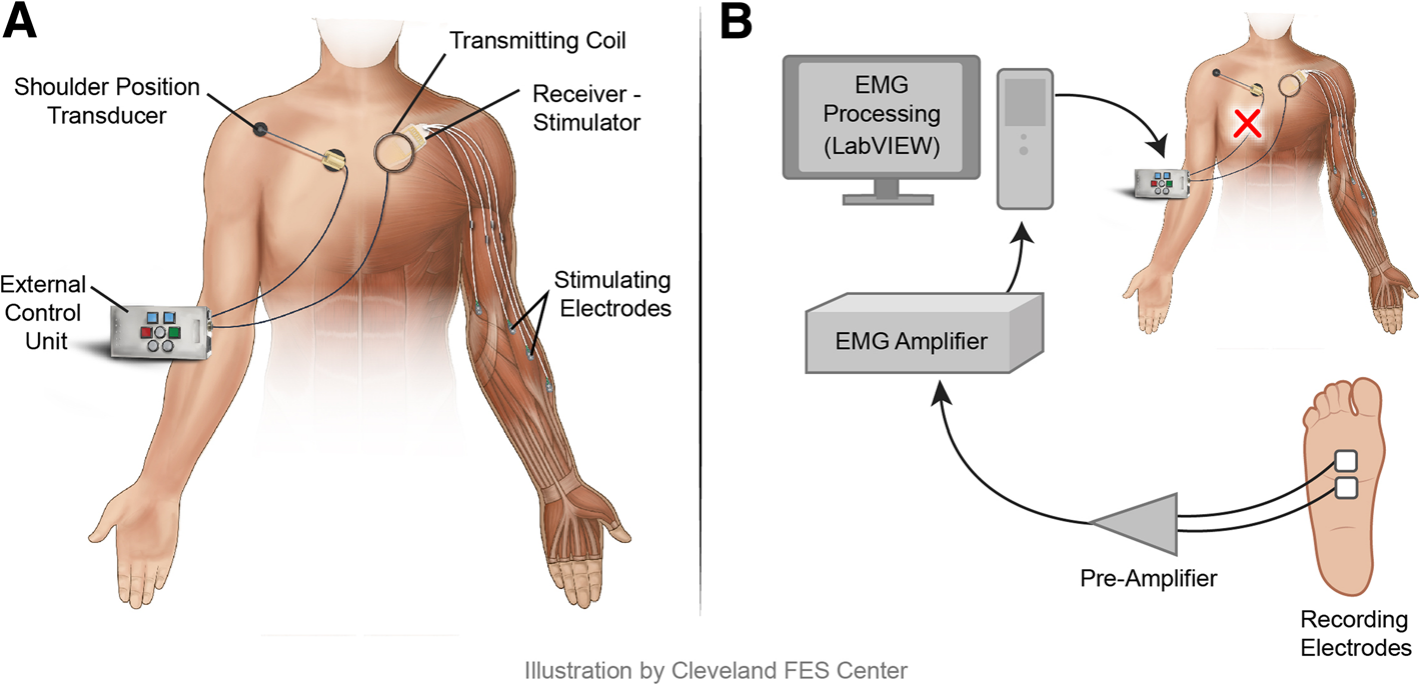
**a**) Typical neuroprosthesis configuration with shoulder position controller. **b**) Configuration used with EMG control. The shoulder position controller was disconnected from the ECU and EMG activity from the subject’s left FDB muscle was substituted as a command signal in its place

The participant (C6, AIS B) has used a Freehand® implanted hand grasp neuroprosthesis since 1996. The neuroprosthesis includes a receiver-stimulator implanted in the chest and eight epimysial electrodes implanted over muscles in the forearm and hand [5]. An external control unit (ECU), powers the implant, processes the command signal input, and sends the appropriate stimulation parameters to be telemetered using radio frequency signals to the implanted receiver-stimulator via a coil taped over the implant location. The command signal comes from an external shoulder position transducer, which proportionally controls the degree of hand opening and closing (Fig. 1a).

The shoulder position controller produces a signal level from 0% (shoulder at rest) to 100% (shoulder elevated), which in turn controls stimulation levels to specific muscles to produce a coordinated grasp [6]. At 0% command, finger extensors are stimulated to hold the hand open. As the shoulder elevates and the command signal progresses from 0 to 100%, the stimulation to these extensors is ramped down, and stimulation to the finger flexors is ramped up [7]. The rate at which each muscle is ramped up/down is specific to each individual user and is dependent on grasp pattern. As an example, for this participant’s lateral grasp pattern, the extensor pollicis longus and extensor digitorum would be receiving full stimulation at 0% command and no stimulation at 100% command; conversely the adductor pollicis, flexor pollicis longus, flexor digitorum superficialis, flexor digitorum profundus, and extensor carpi ulnaris are not stimulated at 0% command and are fully stimulated at 100% command.

Assessment of EMG from muscles below the level of injury was performed with this participant as previously described [3]. Testing revealed that this participant could generate a repeatable EMG signal on command from his left flexor digitorum brevis (FDB) muscle, despite the lack of any visual evidence of muscle activity. As shown in Fig. 1b, the EMG signal from the FDB was used to control the opening and closing of the hand via electrical stimulation. EMG recorded from bipolar surface electrodes was rectified, averaged, and scaled by a custom software program in LabVIEW (National Instruments, Austin, TX). An adaptive step size filter [8] processed the signal into a proportional command between 0 and 100%, with 0 and 100% corresponding to stimulation levels positioning the hand completely open or closed respectively, in the same manner as described above for the shoulder position controller. To evaluate performance, first, a grasp force transducer [9] was used to demonstrate correlation of the EMG signal and grasp output. For this evaluation, the four fingers were placed around the sensor opposite the thumb. The palmar grasp pattern was selected, and the subject squeezed the sensor, using his FDB EMG to control the grasp. The EMG signal, the command output, and the grasp sensor voltage were recorded simultaneously.

Secondly, functional performance using the FDB-controlled grasp was directly compared to the subject’s shoulder position-controlled grasp, using the pre-test portion of the Grasp-Release Test (GRT). [10, 11]. The GRT evaluates which of the six objects (peg, block, video tape, fork, can, weight) the subject is able to grasp, move and release in 30 s [10]. The test was performed the neuroprosthesis off, with the neuroprosthesis using the typical shoulder control, and with the neuroprosthesis using FDB EMG control. For this testing, both lateral and palmar grasp patterns were used, depending on the object and selected by the user, however the chosen pattern was consistent for each object between control methods. The protocol was approved by the local Institutional Review Board.

## Results

**Fig. 2:**
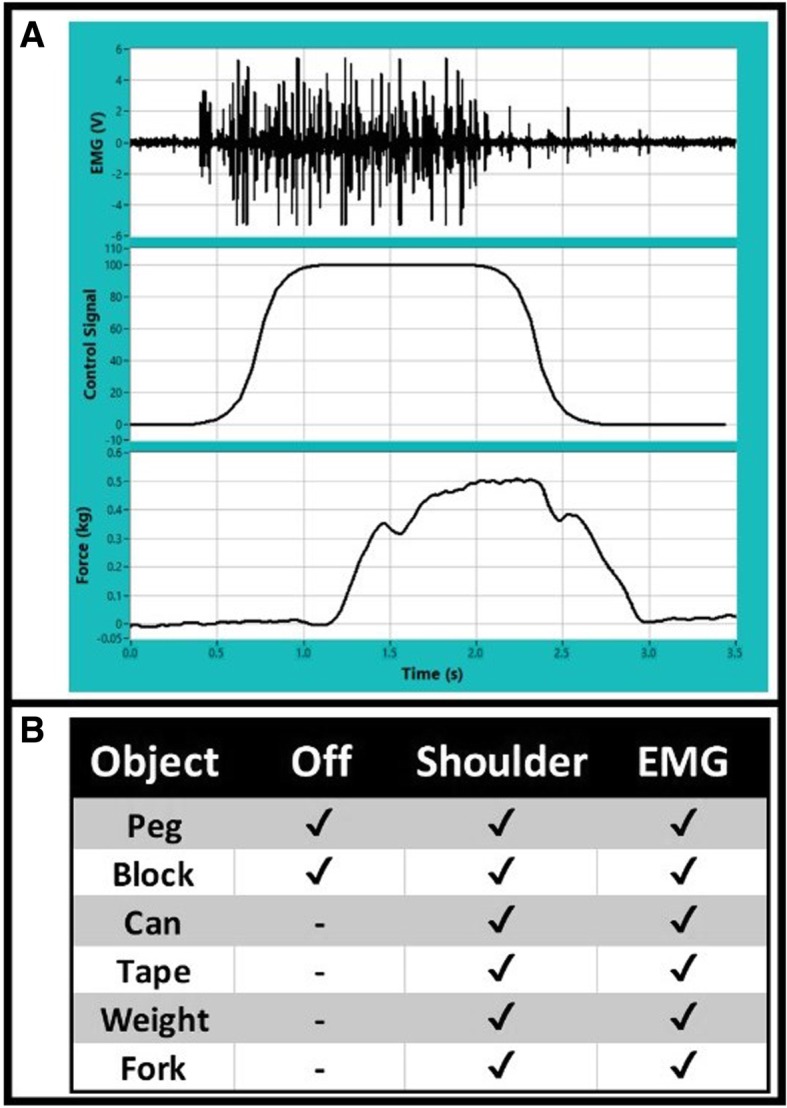
**a**) Traces showing correlation between raw EMG (top), command signal (middle) and grasp force (bottom). **b**) Results from GRT pre-test showing object completions for each control method

The subject was able to open and close his hand using the volitional FDB EMG as a command source. Figure 2a shows the translation of EMG to grasp. The top trace shows full-bandwidth EMG activity. The signal is rectified, averaged, and adaptively filtered to produce the command signal between 0 and 100 shown in the middle trace. As the command signal increases, the hand closes, and once the hand is completely closed, force increases, as shown in the bottom trace. The small delay seen between the command signal and the force output is inherent to muscle stimulation used in NP systems.

Functionally, the participant had the same success in the GRT pre-test using FDB EMG control as he did using his shoulder controller (Fig. 2b). He was able to successfully complete the fork, weight, can, and tape using control from his foot muscle; all objects he could not complete without his neuroprosthesis. He is able to move light objects on his own, and thus could complete the peg and block both with and without the neuroprosthesis.

## Discussion

This is the first demonstration of using muscle activity from below the injury level in a human subject with a clinically complete SCI to command a device that restores function. Here we have shown that functional below-injury control options can exist below the injury level and that this option should not be overlooked, especially in the case where above-injury control options may be limited.

While the participant in this report still uses a shoulder position controller, newer iterations of implanted motor neuroprostheses are controlled through EMG from above-injury muscles under volitional control [12]. Though it has previously been shown that this myoelectric control and shoulder position control are not significantly different in terms of functional performance [8], myoelectric control has an advantage in that it can be implemented without donning/doffing external sensors, it allows more flexible control algorithms, and due to ipsilateral control it makes feasible bilateral grasp systems [2]. Whenever possible, control muscles have been chosen that are synergistic to the function being controlled, i.e. a wrist extensor is preferred to proportionally control a grasp function. This synergistic control is easy for users to learn [13], however, synergists are not available for all functions that could be restored, and thus the control scheme is augmented by using signal from another muscle as a logical switch, such as to trigger function, lock position, or enter a different functional mode. These logical commands are often distinguished from each other by features such as number of consecutive twitches, or the duration the signal is held above a high threshold [2].

As more control tasks are assigned to a single muscle and the control scheme becomes more complex, the user is less likely to remember or use all available functions of the system. Rather than assigning many control tasks to one or two control muscles, the complexity and cognitive burden of using the system could be reduced by implementing a greater number of control signals that each control a single degree of freedom. Unfortunately, the number of above-injury muscles that are available to produce command signals is often fewer than desired. Subsequently, the functions provided to the user are only a subset of the functions we have the capability to provide. In our experience, for example, C6 NP users are practically limited to only a few control source muscles: a wrist extensor, the platysma, and the trapezius. The use of other muscles is not ideal, with upper arm and shoulder muscles being too critical in all arm movements to be given additional control tasks, and facial muscles being undesirable because of the expressions they produce or because they are needed for other activities (eating, talking, etc.). For weaker users (C5 or higher), the control options rapidly dwindle, as a wrist extensor is not available, and this problem is compounded by the fact that these weaker subjects need control over even more degrees of freedom. This lack of available control muscles limits the functions, in number, complexity, and efficiency, which can be effectively provided to the NP user. Using below-injury muscles as control sources is one option to increase the number of available control signals and therefore increase the function provided to the user. For example, rather than needing to toggle a hand system between grasp open/close mode and hand pronation/supination mode, a below-injury myoelectric signal could be used to supplement the system and provide independent control over pronation/supination without switching system modes.

For the purposes of neuroprosthetic control, the authors are not suggesting that above-injury control methods be replaced with below-injury control; nor can we conclude that this option is superior to above-injury control; rather, the purpose of this case report was to present a novel option for control. It is likely that, ultimately, a combination of above- and below-injury controls may provide the optimal control scheme in a multifunction device – this is an area for future study.

It is possible that below-injury control may be less intuitive, and require a higher level of cognitive load, than control above the injury level. In this study, we did not attempt to quantify cognitive load or learning patterns of using below-injury control as compared to traditional control. Quantifying these differences would be an area for future study. Anecdotally, the participant, who had used the shoulder position controller daily for over 20 years, was able to adjust to the FDB control within a few minutes. While he said he preferred using his traditional shoulder control as he was more familiar with it, he also indicated that, were it available in a take-home version, he did not believe using FDB control outside of the lab would be particularly difficult.

Another area to explore in future studies is the possibility of control signals from distal upper-extremity muscles below the injury level, as opposed to lower extremity signals. In our previous study [4], we chose only to screen for signals in the lower extremity, as it is theorized that axons leading to distal leg musculature may be more likely to be spared due to the somatotopic organization of the tracts in the spinal cord. Additionally, screening for volitional signals using surface EMG from muscles just below the injury level becomes technically difficult due to cross-talk from muscles still under volitional control. However, if upper-extremity below-injury controls do exist, they may provide more intuitive control than a lower-extremity muscle. Work done by Pierella et al. [14] presents 2 participants with SCI, 6 months post-injury, who were able to recruit more distal upper-extremity muscles after a rehabilitative training paradigm, based on inertial sensors worn on the upper extremity, was tuned to encourage this movement over more proximal movements. These results are promising that there may also be untapped potential for control signals in distal upper-limb muscles.

This study opens up several other areas for further exploration. Though this study focused on a single muscle, multiple muscles may be viable sources of control information. As more control signals are added to a system, investigating cognitive load and usability will become increasingly important. A more robust study of signal properties, such as amplitude resolution (ability of user to achieve and maintain signal levels between 0 and 100%), and temporal properties (time it takes to turn signal on or off; maximum duration of “on” signal) will be important for these type of signals to be permanently designated as device controls. However, those properties will likely vary between signals, and control requirements will likewise vary based on the function being controlled. In-depth analysis of the signal properties is the subject of future work. Additionally, though no training was required for this specific muscle, training may be able to improve muscle signal strength and other signal properties. A current study is underway to investigate training effects.

The results presented here show the functional impact that even a small amount of residual axonal sparing can provide. This could lend new context to the results of various clinical trials implementing neuroprotective or regeneration therapies, significantly reducing the amount of recovery needed to lead to a functional outcome.

Though this study showed a simple proof-of concept, the idea is feasible as part of a fully implanted, clinically deployed system, using current modular neuroprosthesis technology [15, 16]. The work presented here is a step toward our end goal of improving the performance of implanted neuroprosthesis to restore function and independence for people with SCI.

## Data Availability

The datasets used and/or analysed during the current study are available from the corresponding author on reasonable request.

## Abbreviations

AIS: ASIA [American Spinal Injury Association] Impairment Scale
ECU: external control unit
EMG: electromyography; electromyographic
FDB: flexor digitorum brevis
GRT: Grasp-Release Test
NP: neuroprosthesis
NPs: neuroprostheses
SCI: spinal cord injury
